# Serum 25-hydroxyvitamin D is negatively associated with severe periodontitis: a cross-sectional study

**DOI:** 10.1186/s12903-021-01850-3

**Published:** 2021-09-27

**Authors:** Fangjing Zhou, Ning Ma, Ruiting Su, Xiaoyu He, Xiaona Wang, Yang Zhou, Jing Shi

**Affiliations:** 1 Shanxi Medical University School and Hospital of Stomatology, Taiyuan, 030012 Shanxi Province China; 2 Department of Emergency Medicine, The Second Xiangya Hospital of Central South University, 139 Renmin road, Changsha, 410011 Hunan Province China; 3 Department of Oral Medicine, The Fifth Clinical Medical College of Shanxi Medical University, 22 Shuangtasi Street, Taiyuan, 030012 Shanxi Province China

**Keywords:** 25-hydroxyvitamin D, Vitamin D, Periodontitis, Periodontal disease, NHANES

## Abstract

**Background:**

Periodontitis can lead to the destruction of periodontium and adversely influence the overall health, wellbeing, and quality of life. However, studies on the relationship between severe periodontitis and serum 25-hydroxyvitamin D [25(OH)D] are limited. This study is designed to explore the relationship between 25(OH)D and severe periodontitis.

**Methods:**

A cross-section study of 2928 participants enrolled from the National Health and Nutrition Examination Survey (NHANES) from 2013 to 2014 was conducted. The periodontal examination was performed using a total oral periodontal examination program, and probe measurements were collected at six sites per tooth in NHANES. Severe periodontitis was characterized as: ≥ 2 interproximal sites with attachment loss (AL) ≥ 6 mm (not on the same tooth) and ≥ 1 interproximal site with probing depth (PD) ≥ 5 mm. Severe periodontitis and serum 25(OH)D were the dependent and independent variables, respectively. Univariate, multivariate, and subgroup analyses were performed to explore the relationship between severe periodontitis and serum 25(OH)D.

**Results:**

Among the 2928 participants, the average age of the population was 50 ± 13.71 years old, with 1425 (48.67%) males, 316 (10.79%) exhibited severe periodontitis. Serum 25(OH)D showed a significantly negative association with severe periodontitis after adjusting all variables (OR 0.75, 95% CI 0.63–0.89). In addition, severe periodontitis has a nonlinear relationship with serum 25(OH)D, whoes inflection point was 102 (nmol/L). On the left side of the inflection point (25(OH)D ≤ 102 nmol/L), the effect size was 0.98 and 95%CI was 0.98–0.99 (25(OH)D per 1 nmol/L increments). On the right side of the inflection point (25(OH)D > 102 nmol/L), the effect size was 0.99 and 95% CI was 0.98–1.01. The subgroup analysis showed pronounced changes in non-Hispanic white, alcohol consumption, diabetes, and health insurance.

**Conclusion:**

Serum 25 (OH) D in relation to severe periodontitis is nonlinear in our study.When serum 25 (OH) D is less than 102 nmol/L, serum 25 (OH) D is negatively associated with severe periodontitis.

**Supplementary Information:**

The online version contains supplementary material available at 10.1186/s12903-021-01850-3.

## Background

Periodontitis is the sixth most prevalent disease globally with significant socioeconomic and systemic repercussions and is one of the most common chronic inflammatory diseases in adults [1]. An estimated 42% of the United States (U.S.) adults aged 30 years and over suffer from periodontal disease [2]. Periodontitis can lead to the destruction of periodontium and adversely influence the overall health, wellbeing, and quality of life [3, 4].

Recent studies have reported an increasing relationship between periodontitis and diabetes, cardiovascular disease, inflammatory bowel disease, sleep disorders, chronic obstructive pulmonary disease, and other systematic diseases [5–7]. Periodontitis is a chronic inflammation disease caused by oral bacterial infection [8]. The body’s immune response to the infection causes damage to the tissue. Vitamin D is essential to the general health, particularly in children, pregnancy, certain forms of cancer, and prevention of infection [9, 10]. Moreover, studies indicated that serum Vitamin D deficiency is related to the severity and progression of periodontal disease [5, 11, 12]. Periodontitis is a multifactorial disease that requires immune and complex infectious interactions [13].

In epidemiological studies, vitamin D supplement was inversely correlation with the incidence of periodontitis [14]. Studies have shown that 25 (OH) D status could alter the risk for periodontitis by regulating the host’s immune response to infection or by preventing alveolar bone loss [8]. Vitamin D is the general term for vitamin D2 and vitamin D3. The former is produced by ultraviolet radiation of yeast ergosterol, while the latter is produced by the ultraviolet radiation of 7-dehydrocholesterol in lanolin, has the biological activity of gallbladder calcium, and is synthesized in human skin [5]. A series of literatures have shown that 1,25-dihydroxyvitamin D3 could inhibit cytokine production and antigen-induced T cell proliferation [15]. However, there is limited clinical studies on the anti-inflammatory effects. Furthermore, Luo et al. found that the second highest level of vitamin D supplement have a lower severity of periodontitis compared to the highest level of vitamin D supplement [14]. It suggested that the correlation between periodontitis and vitamin D may be nonlinear. Recently, evidence indicated that high serum vitamin D level would protect individuals from oral diseases [12, 16]. However, the nonlinearity was not explored in their researches. Thus, the hypothesis of this study was that the relationship between serum vitamin D and severe periodontitis may be non-linear.

Hence, this study aims to clarify the association of 25 (OH) D and severe periodontitis using a typical sample. The data were obtained from the NHANES from 2013 to 2014. The datasets from this period were favorable as they contained information on the latest periodontal examination, as well as severe periodontitis.

## Methods

NHANES protocol approved by NCHS Research Ethics Review Board, and obtained informed consent from all participants (https://www.cdc.gov/nchs/nhanes/index.htm). The Institutional Review Board at the Shanxi Provincial People's Hospital determined the analysis used public data sets that did not constitute human subjects research, so human subjects approval was not needed.

### Study design and participants

This is a cross-sectional study. The study was organized based on the list of items for the STROBE (cross-sectional studies). Trained staff conducted examinations and interviews through automated data collection where questions were sent directly to the participants or through an agent if necessary. Individuals were enrolled in NHANES data from 2013 to 2014. Inclusion criteria included participants with complete full-mouth periodontal examination (FMPE). Exclusion criteria included: (1) uncompleted with 25-hydroxyvitamin D tests; (2) aged less than 30 years old. A total of 10,175 individuals were screened, out of which 2928 individuals were sampled for the interviews.

### Primary variables

Periodontal examination applied the FMPE protocol, which collects probe measurements from six sites per tooth for all teeth in NHANES. Accordingly, the CDC/AAP definitions were based on measures of AL and PD at the four interproximal sites per tooth [17] Severe periodontitis was characterized as: ≥ 2 interproximal sites with attachment loss (AL) ≥ 6 mm (not on the same tooth) and ≥ 1 interproximal site with probing depth (PD) ≥ 5 mm. Mild periodontitis, moderate periodontitis, and no periodontitis definitions were described in Additional file 1: Table S1.

Using the standard protocol, serum 25(OH)D levels were tested in blood samples collected through venipuncture at mobile examination centers (MEC). Serum 25(OH)D was measured via DiaSorin radioimmunoassay kit (Diasorin, Stillwater, MN, USA) at the National Center for Environmental Health, Center for Disease Control and Prevention. As in previous studies, the serum 25(OH)D levels were classified as normal (≥ 75 nmol/ml), insufficient (50–74.9 nmol/ml), deficient (25–49.9 nmol/ml), and severely deficient (< 25 nmol/ml) [16].

### Study variables

Potential confounding factors were selected from previous studies, including gender, race, body mass index (BMI), age, education, diabetes mellitus, marital status, smoking status, hypertension, poverty-income ratio (PIR), alcohol consumption, hypercholesterolemia, triglyceride, apolipoprotein (B), health insurance, total cholesterol, low-density lipoprotein cholesterol (LDL-c), high-density lipoprotein cholesterol (HDL-c), glycohemoglobin, and fasting blood glucose. The definitions of smoking status and alcohol consumption were based on our previous study (Additional file 1) [16]. Patients were considered hypertensive based on a medical report or if they were on antihypertensive drugs. The diagnosis of hypercholesterolemia was based on cholesterol test results or if patients were on lipid-lowering drugs. The race was classified as other race, non-Hispanic white, non-Hispanic black, other Hispanic, or Mexican–American. Health insurance status was captured by the question: “Do you have health insurance or other kinds of health insurance?” As mentioned earlier, the household PIR is obtained by dividing the household income by the poverty criterion. A college degree or above, certain college or associate (AA) degrees, a high school graduation, or below 11th grade are classified based on educational levels. The marital status was categorized into married, separated, never married, living with a partner, divorced, or widowed.

### Statistical analysis

Baseline characteristics were analyzed using means, standard errors (SE), median (min–max), percentages, or frequencies. Continuous variables were compared using analysis of variance (ANOVA) for normally distributed variables, and non-parametric test for not fit the normal distribution. Categorical variables were analyzed using the Chi-square test. We adjusted the *p* value of multiple groups testing to perform the large number of tests by Bonferroni correction. The effect of 25(OH)D on the severe periodontal disease was examined using multiple logistic regression model. Model I: no adjustment, Model II: adjusted for gender and age, and Model III: adjusted for age, BMI, hypertension, gender, marital status, alcohol consumption, education level, race/Hispanic origin, apolipoprotein (B), hypercholesterolemia, glycohemoglobin, HDL-C, health insurance, total cholesterol, triglyceride, LDL-C, and smoking status. Further, converting classification variables of 25(OH)D to continuous variables to perform tests for linear trend for robust analysis in the models. To account for 25(OH)D as a continuous variable, the effect value for severe periodontitis per increase in 25(OH)D per standard deviation (SD) was determined. a smooth curve fitting and a generalized additive model was established to address the association of severe periodontitis and 25(OH)D. When nonlinearity is detected, the recursive algorithm is first used to calculate the inflection point, and then a two-segment linear regression model is built on both sides of the inflection point. Interaction and stratified analyses were performed based on all variables outlined in Table 1. Subgroup analysis was performed to assess the potential effect of unmeasured confounding variables. R (http://www.R-project.org) and Empower-Stats were used for all analyses. Two-sided *p* values < 0.05 were considered statistically significant.

## Results

**Fig. 1:**
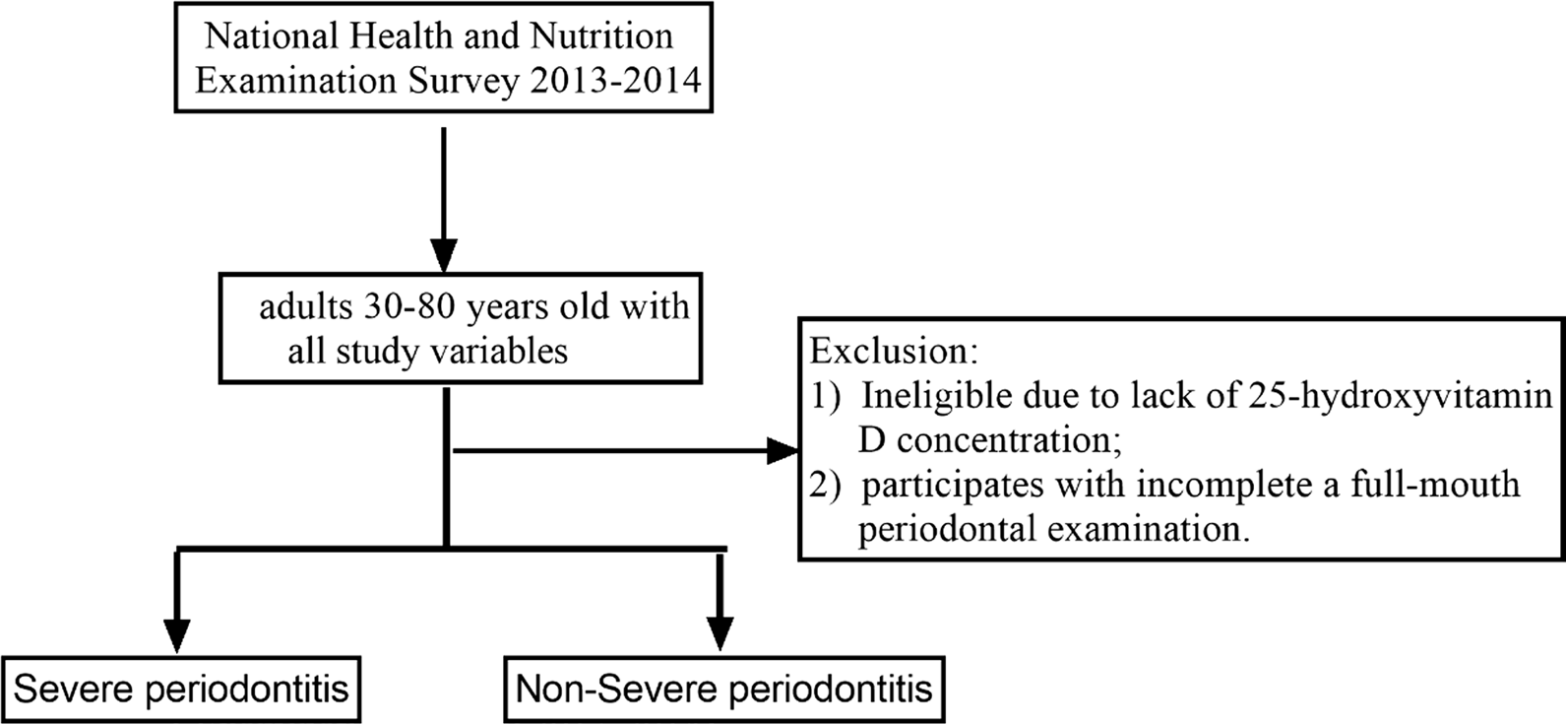
Flow chat of sample selection from NHANES 2013 to 2014

A total of 2928 participants were enrolled in our study (Fig. 1). 10.79% (n = 316) of the participants had severe periodontitis.

Table 1 shows sociodemographic characteristics and other covariates from NHANES (2013–2014). The population mean age was 50 ± 13.71 (30–80) years old, with 1425 (48.67%) males. Glycohemoglobin, apolipoprotein (B), fasting glucose, total cholesterol, LDL-C, triglyceride, and diabetes mellitus were similar in the different groups of serum 25(OH)D. Compared with the deficient, insufficient, and normal groups, individuals with severely deficient 25(OH)D were likely to have severe periodontal disease, high creatinine, BMI, PIR, alcohol consumption, live single/divorced/widowed, and non-Hispanic black. An opposite pattern was observed for age, HDL-C, education level (college graduate or above), hypercholesterolemia, and current smoking.

**Table 1:** Baseline characteristics of the participates

Characteristics	Serum 25-hydroxyvitamin D, nmol/L			
	Severely deficient (< 25)	Deficient (25–49.9)	Insufficient (50–74.9)	Normal (≥ 75)
N	105	697	1104	929
Age(year)	**46.03 ± 11.88**	**46.20 ± 11.95**	**48.48 ± 12.81**	**55.14 ± 14.61**
Gender				
Male	**46 (43.81%)**	**352 (50.50%)**	**584 (52.90%)**	**389 (41.87%)**
Female	**59 (56.19%)**	**345 (49.50%)**	**520 (47.10%)**	**540 (58.13%)**
Creatinine, urine(umol/L)	**10,961 (6806, 16,663)**	**10,210 (5569, 1547)**	**9370. (5304, 14,409)**	**7602 (4243, 12,287)**
Apolipoprotein (B) (mg/dL)	90.44 ± 20.80	93.97 ± 24.13	94.20 ± 26.18	92.33 ± 25.44
BMI(Kg/m2)	**33.19 ± 8.83**	**30.87 ± 7.61**	**29.07 ± 6.18**	**27.96 ± 6.51**
Glycohemoglobin (%)	5.89 ± 1.26	5.83 ± 1.21	5.70 ± 0.94	5.70 ± 0.96
Fasting Glucose (mmol/L)	6.00 ± 2.36	6.10 ± 2.11	5.97 ± 1.80	5.83 ± 1.49
Direct HDL-Cholesterol (mmol/L)	**1.34 ± 0.39**	**1.30 ± 0.39**	**1.33 ± 0.39**	**1.50 ± 0.46**
Total Cholesterol (mmol/L)	4.85 ± 0.90	5.03 ± 1.13	5.02 ± 1.00	5.06 ± 1.18
Triglyceride (mmol/L)	**1.01 (0.71, 1.42)**	**1.03 (0.72, 1.67)**	**1.17 (0.78–1.82)**	**1.06 (0.74, 1.53)**
LDL-cholesterol (mmol/L)	2.85 ± 0.73	3.00 ± 0.87	3.00 ± 0.92	2.98 ± 0.91
Race/Hispanic origin				
Mexican American	**12 (11.43%)**	**144 (20.66%)**	**184 (16.67%)**	**75 (8.07%)**
Other Hispanic	**6 (5.71%)**	**59 (8.46%)**	**126 (11.41%)**	**51 (5.49%)**
Non-Hispanic White	**14 (13.33%)**	**172 (24.68%)**	**467 (42.30%)**	**589 (63.40%)**
Non-Hispanic Black	**59 (56.19%)**	**198 (28.41%)**	**147 (13.32%)**	**95 (10.23%)**
Other Race	**14 (13.33%)**	**124 (17.79%)**	**180 (16.30%)**	**119 (12.81%)**
Education level				
Less than 11th grade	**19 (18.10%)**	**159 (22.81%)**	**221 (20.02%)**	**117 (12.61%)**
High school graduate/GED	**28 (26.67%)**	**154 (22.09%)**	**224 (20.29%)**	**170 (18.32%)**
Some college or AA degree	**36 (34.29%)**	**205 (29.41%)**	**316 (28.62%)**	**290 (31.25%)**
College graduate or above	**22 (20.95%)**	**179 (25.68%)**	**343 (31.07%)**	**351 (37.82%)**
Marital status				
Single/divorced/widowed	**31 (29.52%)**	**142 (20.37%)**	**216 (19.58%)**	**205 (22.07%)**
Never married	**26 (24.76%)**	**115 (16.50%)**	**96 (8.70%)**	**85 (9.15%)**
Married/living as married	**48 (45.71%)**	**440 (63.13%)**	**791 (71.71%)**	**639 (68.78%)**
Ratio of family income to poverty				
< 1.3	**41 (40.20%)**	**231 (36.09%)**	**270 (26.63%)**	**185 (21.54%)**
1.3–3.5	**44 (43.14%)**	**220 (34.38%)**	**357 (35.21%)**	**253 (29.45%)**
> 3.5	**17 (16.67%)**	**189 (29.53%)**	**387 (38.17%)**	**421 (49.01%)**
Alcohol consumption				
No	**21 (21.00%)**	**188 (29.15%)**	**242 (23.16%)**	**226 (25.54%)**
Yes	**79 (79.00%)**	**457 (70.85%)**	**803 (76.84%)**	**659 (74.46%)**
Hypertension				
No	**62 (59.05%)**	**459 (65.85%)**	**777 (70.38%)**	**535 (57.65%)**
Yes	**43 (40.95%)**	**238 (34.15%)**	**327 (29.62%)**	**393 (42.35%)**
Hypercholesterolemia				
No	74 (70.48%)	484 (69.44%)	693 (62.77%)	503 (54.14%)
Yes	31 (29.52%)	207 (29.70%)	404 (36.59%)	425 (45.75%)
Diabetes mellitus				
No	91 (86.67%)	625 (89.80%)	983 (89.04%)	812 (87.50%)
Yes	14 (13.33%)	71 (10.20%)	121 (10.96%)	116 (12.50%)
Health insurance				
No	**28 (26.67%)**	**204 (29.27%)**	**242 (21.94%)**	**106 (11.41%)**
Yes	**77 (73.33%)**	**493 (70.73%)**	**861 (78.06%)**	**823 (88.59%)**
Smoking status				
Never smoking	**54 (51.43%)**	**406 (58.33%)**	**646 (58.51%)**	**562 (60.56%)**
Former smoking	**34 (32.38%)**	**135 (19.40%)**	**147 (13.32%)**	**100 (10.78%)**
Current smoking	**17 (16.19%)**	**155 (22.27%)**	**311 (28.17%)**	**266 (28.66%)**
Severe periodontitis				
No	**81 (77.14%)**	**596 (85.51%)**	**997 (90.31%)**	**860 (92.57%)**
Yes	**24 (22.86%)**	**101 (14.49%)**	**107 (9.69%)**	**69 (7.43%)**

Bold denotes statistical signifcance at p < 0.05

*p* values were calculated using the Chi-square, analysis of variance (ANOVA) and Bonferroni correction

BMI, body mass index; AA, associate degree; GED, General Educational Development; HDL-Cholesterol, high-density lipoprotein cholesterol; LDL-Cholesterol, low-density lipoprotein cholesterol

**Fig. 2:**
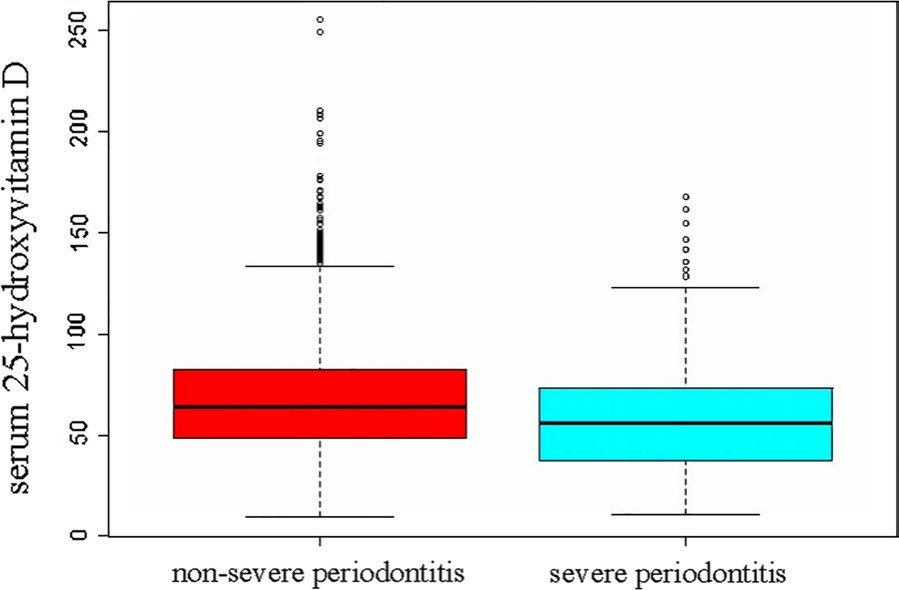
Comparison of serum 25-hydroxyvitamin D value between non-severe periodontitis and severe periodontitis group by non-parametric test (F = 27.9, p < 0.05). The bottom and top edges of each box represent the first and third quartiles, respectively, the band within the box represents the median value

The univariate analysis suggested that age, female, race, education level, marital status, PIR, hypertension, glycohemoglobin, health insurance, and smoking status were significantly associated with the severe periodontitis (Additional file 1: Table S2). Also, 25(OH)D were increased in the non-severe periodontitis than severe periodontitis group (Fig. 2).

**Fig. 3:**
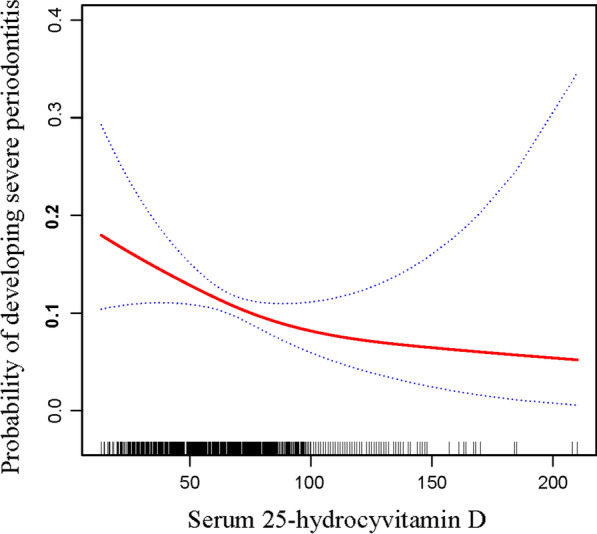
The correlation between serum 25-hydroxyvitamin D and severe periodontitis. Association between serum 25-hydroxyvitamin D and severe periodontitis. A threshold, nonlinear association between serum 25-hydroxyvitamin D and severe periodontitis was found in a generalized additive model (GAM, *P* < 0.01). Red line represents the smooth curve fit between variables. Blue lines represent the 95% of confidence interval from the fit

Table 2 shows serum 25(OH)D is related to severe periodontitis at three models. The results show that per SD increase in serum 25(OH)D levels is related to develop severe periodontitis (OR 0.75, 95% CI 0.63–0.89). An increase in 25(OH)D levels was associated with a reduced probability of developing severe periodontitis (OR 0.75, 95% CI 0.63–0.89). In Model I, which was unadjusted, 25(OH)D was negatively associated with severe periodontitis (OR 0.69, 95% CI 0.60–0.79). After adjusting for age and gender in Model II, similar results were detected (OR 0.57, 95% CI 0.49–0.66). After adjusting significant variables in Additional file 1: Table S2, an increase in 25(OH)D level was also negatively associated with severe periodontitis (OR 0.75, 95% CI 0.63–0.89). Taken together, these findings indicated that serum 25(OH)D levels were significantly associated with reduced risk of severe periodontal disease. Therefore, we also established a generalized additive model and a smooth curve fitting to assess the relation between severe periodontitis and 25(OH)D (Fig 3). The result indicates that the two piecewise linear regression was more suitable for fitting the association between 25(OH)D and severe periodontitis because it can accurately represent the relationship between severe periodontitis and 25(OH)D. We calculated the inflection point was 102 (nmol/L) through the two-piecewise linear regression and recursive algorithm. On the left side of the inflection point (25(OH)D ≤ 102 nmol/L), the effect size was 0.98 and 95%CI was 0.98–0.99 (25(OH)D per 1 nmol/L increments). On the right side of the inflection point (25(OH)D > 102 nmol/L), the effect size was 0.99 and 95% CI was 0.98–1.01 (Table 3).

**Table 2:** Multiple logistic regression model for the association between 25(OH)D (nmol/L) and severe periodontitis in different models

Exposure	Model I		Model II		Model III	
	OR, 95%CI	*p* value	OR, 95%CI	*p* value	OR, 95%CI	*p* value
Serum 25(OH)D, nmol/L						
Severely deficient (< 25)	Reference		Reference		Reference	
Deficient (25–49.9)	0.57 (0.35, 0.94)	0.029	0.51 (0.30, 0.86)	0.011	0.68 (0.37, 1.24)	0.208
Insufficient (50–74.9)	0.36 (0.22, 0.60)	< 0.001	0.27 (0.16, 0.45)	< 0.001	0.58 (0.31, 1.08)	0.086
Normal (≥ 75)	0.27 (0.16, 0.45)	< 0.001	0.16 (0.09, 0.28)	< 0.001	0.36 (0.19, 0.69)	0.002
P for trend	< 0.001			< 0.001		
Serum 25(OH)D, per SD	0.69 (0.60, 0.79)	< 0.001	0.57 (0.49, 0.66)	< 0.001	0.75 (0.63, 0.89)	0.001

Model I: adjust for none

Model II: adjust for gender, age

Model III: adjust for gender; hypertension; direct HDL-Cholesterol, age, race/Hispanic origin, glycohemoglobin, education level, diabetes mellitus, BMI, marital status, creatinine, health insurance, smoking status, ratio of family income to poverty

**Table 3:** The results of the two-piecewise linear model

	Severe periodontitis (OR, 95%CI)	*p* value
Fitting model by standard linear regression, per SD increments	0.75 (0.64, 0.89)	0.001
Fitting model by two-piecewise linear regression		
Inflection point of lactate dehydrogenase, nmol/L	102	
≤ 102	0.98 (0.98, 0.99)	0.001
> 102	0.99 (0.98, 1.01)	0.798

Adjusted: gender; hypertension; HDL-Cholesterol, age, race/Hispanic origin, glycohemoglobin, education level, diabetes mellitus, BMI, marital status, creatinine, health insurance, smoking status, ratio of family income to poverty

BMI, body mass index; HDL, high-density lipoprotein; SD, standard deviation

Subgroup analysis results are shown in Additional file 1: Table S3. Race, alcohol consumption, diabetes mellitus, and health insurance were potential confounders of 25(OH)D and severe periodontitis (*P* < 0.05).

## Discussion

This study is one of the largest epidemiologic studies to investigate the association between serum 25(OH)D and severe periodontitis. After adjusting for confounders in the full adjusted model, we found that 25(OH)D is negatively associated with severe periodontitis. The increase in per SD serum 25(OH)D levels corresponded to a 25% decrease in the probability of severe periodontitis. Besides, a non-linear relationship was detected between severe periodontitis and serum 25(OH)D. On the left side of the inflection point, the risk of perodontitis in U.S. participants was reduced by 2% for each additional 1 nmol/L of serum 25(OH)D. On the right side of the inflection point, the relationship cannot be observed (95% CI was 0.99, 0.98–1.01, *P* = 0.798). Besides, there was a stronger association between 25(OH)D and severe periodontitis in non-Hispanic white, alcohol consumption, diabetes, and health insurance populations after subgroup analysis.

Previous studies showed that the serum 25(OH)D level or Vitamin D intake were related to periodontitis [12, 18, 19]. However, some studies reported no disparity between 25(OH)D and periodontal disease severity [5, 20]. A cohort study using NHANES III data found 25(OH)D is not associated with periodontitis in matched populations younger than 50 years old [21]. Besides, NHANES III data were limited to sites without any attachment loss due to periodontitis. Recent studies indicated that the level of vitamin D intake is related to periodontitis using recently NHANES data (2013–2014) [14]. NHANES data (2013–2014) and the periodontal examination was based on FMPE protocol, which was more accurate than before. Our study is similar with previous studies that serum 25(OH)D were also related with severe periodontitis. Recently, a series of studies reported that serum 25(OH)D was associated with periodontitis [2, 12, 22]. However, nonlinearity and subgroup analysis were not performed in their researches. Therefore, the contribution of this study was the discovery of a non-linear and threshold effect on the relationship between 25(OH)D and severe periodontitis. The immune and inflammatory response against periodontal pathogens are suggested to be triggered by the host immune system [23]. Moreover, some results have shown that during severe periodontitis, vitamin D receptors in the immune system cells protect periodontal tissue endothelium and reduce the release of B and T lymphocytes caused by periodontal pathogens [24].

Subgroup analysis was used to assess the independent relationship between 25(OH)D and severe periodontitis. Gender, age, race, alcohol consumption, education level, apolipoprotein, BMI, marital status, hypertension, PIR, diabetes, glycohemoglobin, fasting glucose, health insurance, smoking status, total cholesterol, triglyceride, and LDL-C were used as category variables, of which interactions were detected in non-Hispanic white, alcohol consumption, diabetes, and health insurance populations. The reason may explain as people with health insurance pay more attention to oral health. Also, study indicated that diabetes increases the risk of periodontal diseases, also, inflammatory diseases may increase insulin resistance [25]. Moreover, we found the level of serum 25(OH)D in non-Hispanic white people was higher than that in other people, and severe periodontitis was lower than that in other races, which were consistent with previous study [26]. A cross-sectional study found no dependence between alcohol and severe periodontal disease [27]. While alcohol consumption is associated with vitamin D [28], which may affect the periodontitis, similar to our results. Therefore, in American adults, there is an urgent need to prevent alcohol consumption at low levels of serum 25 (OH) D with severe periodontitis.

The present study is of clinical value. To the best of our knowledge, we use the latest data from NHANES to assess the association of serum 25(OH)D levels and periodontitis. The results of this study may be useful for further pathogenesis research. Our research will also contribute to the evidence to the literature.

Our study has some advantages. (1) We address the nonlinear problem in our current study and explore it further. (2) Due to a cross-sectional study; hence, if may be prone to biases. Because we used strict statistical adjustments, the remaining confounding factors were minimized. (3) We convert the target-independent variable into both a categorical and a continuous variable. This method may restrict data analyzed and enhance the robustness of the results. (4) Effect correction factor analysis enabled this study to make better use of the data to produce stable conduction in different subgroups.

This research also has some shortcomings. First, our findings are based on a U.S. population, therefore, these findings may not be extrapolated to other nationalities. Second, some important confounders such as the sunshine time and daily consumption that affect the levels of vitamin D and the inflammatory status were not achieved. Nevertheless, our results indicated 25(OH)D was negatively related to severe periodontal disease. Taking vitamin D or receiving more sunshine may cause the relationship of the smooth curve fitting in Fig. 3 to disappear. Third, due to the definition of non-severe periodontitis include mild and moderate periodontitis patients, which may lead to some selected bias. Forth, cross-sectional studies alone cannot establish a causal connection between severe periodontitis and 25(OH)D. Therefore, well-designed investigates should be explored to determine the potential anti-inflammatory effect of 25(OH)D on periodontitis.

## Conclusion

The relationship between serum 25(OH)D and severe periodontitis in our study population is non-linear. Serum 25(OH)Dwas negatively related to severe periodontitis when it is less 102 nmol/L. The association, however, should be further evaluated using long-term cohort studies.

## Supplementary Information

**Additional file 1: Table S1.** Case definitions proposed for population based surveillance to periodontitis;** Table S2**. Univariate analysis for severe periodontitisUnivariate analysis for severe periodontitis;** Table S3**.  Subgroup analysis for the interaction of 25(OH)D levels in different group subgroup analysis for the interaction of25(OH)D levels in different group.

## Data Availability

The NHANES data of this study are openly available at https://www.cdc.gov/nchs/nhanes/default.aspx.

## Abbreviations

NHANES: National Health and Nutrition Examination Survey
25(OH)D: 25-Hydroxyvitamin D
NCHS: National Center for Health Statistics
FMPE: Full-mouth periodontal examination
BMI: Body mass index
AA: Associate degree
GED: General Educational Development
HDL-Cholesterol: High-density lipoprotein cholesterol
LDL-Cholesterol: Low-density lipoprotein cholesterol
