# Supplementary material for: Serum 25-hydroxyvitamin D is negatively associated with severe periodontitis: a cross-sectional study
Source: BMC Oral Health. 2021 Sep 27;21:479. doi: 10.1186/s12903-021-01850-3 (PMC8477570; doi:10.1186/s12903-021-01850-3)
Supplement: Supplementary file 1 — Additional file 1: Table S1. Case definitions proposed for population based surveillance to periodontitis; Table S2. Univariate analysis for severe periodontitisUnivariate analysis for severe periodontitis; Table S3. Subgroup analysis for the interaction of 25(OH)D levels in different group subgroup analysis for the interaction of25(OH)D levels in different group. [file 12903_2021_1850_MOESM1_ESM.docx]

**Serum 25-hydroxyvitamin D is negatively associated with severe periodontitis: a cross-sectional study**

Fangjing Zhou*, Ning Ma*, Ruiting Su*, Xiaoyu He*, Xiaona Wang^‡^, Yang Zhou^†^, Jing Shi^‡^

* Shanxi Medical University, Taiyuan, Shanxi Province 030012, China

^†^ Department of Emergency Medicine, the Second Xiangya Hospital of Central South University, 139 Renmin road, Changsha, Hunan province, 410011, China

^‡^ Department of Oral Medicine, Shanxi Provincial People's Hospital Affiliated to Shanxi Medical University, 22 Shuangtasi Street, Taiyuan, Shanxi Province 030012, China.

Correspondence should be addressed to Jing Shi, M.D. Ph.D.

**Email:** crystalshi@163.com

**Intitution:** Department of Oral Medicine, Shanxi Provincial People's Hospital Affiliated to Shanxi Medical University, 22 Shuangtasi Street, Taiyuan, Shanxi Province 030012, China.

**Supplement Table 1** Case definitions proposed for population based surveillance to periodontitis^[^[^1^](#_ENREF_1)^]^.

| **Case** | **Definition** |
| --- | --- |
| No periodontitis | No evidence of mild, moderate, or severe periodontitis |
| Mild periodontitis | ≥2 interproximal sites with AL ≥ 2 interproximal sites with PD ≥ 5mm (not on same tooth) |
| Moderate periodontitis | ≥2 interproximal sites with AL ≥ 4mm (not on same tooth), or ≥2 interproximal sites with PD ≥ 5mm (not on same tooth) |
| Severe periodontitis | ≥2 interproximal sites with AL ≥ 6mm (not on same tooth) and ≥ 1 interproximal sites with PD ≥ 5mm |

These definitions are now commonly referred to as the CDC-AAP case definitions for surveillance of periodontitits.

Third molars excluded; total periodontitis is defined as the sum of mild, moderate, and severe disease.

**Reference:** [1] EKE P I, PAGE R C, WEI L, et al. Update of the case definitions for population-based surveillance of periodontitis [J]. Journal of periodontology, 2012, 83(12): 1449-54.

**Supplement Table 2**. Univariate analysis for severe periodontitis.

| Characteristics | Statistics | Periodontitis | *P*-value |
| --- | --- | --- | --- |
| Age(year) | 50.00 ± 13.71 | 1.04 (1.03, 1.04) | <0.001 |
| Gender |  |  |  |
| Male | 1425 (48.67%) | Reference |  |
| Female | 1503 (51.33%) | 0.41 (0.32, 0.53) | <0.001 |
| Race/Hispanic origin |  |  |  |
| Mexican American | 427 (14.58%) | Reference |  |
| Other Hispanic | 253 (8.64%) | 0.75 (0.46, 1.21) | 0.234 |
| Non-Hispanic White | 1267 (43.27%) | 0.48 (0.34, 0.68) | <0.001 |
| Non-Hispanic Black | 525 (17.93%) | 1.60 (1.13, 2.26) | 0.008 |
| Other Race | 456 (15.57%) | 0.47 (0.30, 0.74) | 0.001 |
| Education level |  |  |  |
| less than 11th grade | 534 (18.24%) | Reference |  |
| high school graduate/GED | 589 (20.12%) | 0.72 (0.53, 0.97) | 0.031 |
| some college or AA degree | 875 (29.89%) | 0.29 (0.21, 0.40) | <0.001 |
| college graduate or above | 929 (31.74%) | 0.14 (0.09, 0.20) | <0.001 |
| Marital status |  |  |  |
| single/divorced/widowed | 614 (20.98%) | Reference |  |
| never married | 338 (11.55%) | 1.02 (0.69, 1.50) | 0.923 |
| married/living as married | 1975 (67.48%) | 0.65 (0.50, 0.86) | 0.002 |
| Ratio of family income to poverty |  |  |  |
| <1.3 | 753 (27.89%) | Reference |  |
| 1.3-3.5 | 906 (33.56%) | 0.73 (0.56, 0.96) | 0.024 |
| >3.5 | 1041 (38.56%) | 0.21 (0.15, 0.30) | <0.001 |
| Creatinine, urine(umol/L) | 10400.60 ± 6864.06 | 1.00 (1.00, 1.00) | 0.070 |
| Alcohol consumption |  |  |  |
| no | 694 (25.23%) | Reference |  |
| yes | 2057 (74.77%) | 1.08 (0.81, 1.43) | 0.603 |
| Apolipoprotein (B) (mg/dL) | 93.37 ± 25.25 | 1.00 (1.00, 1.01) | 0.442 |
| BMI(Kg/m2) | 29.30 ± 6.96 | 1.01 (0.99, 1.02) | 0.552 |
| Hypertension |  |  |  |
| no | 1898 (64.87%) | Reference |  |
| yes | 1028 (35.13%) | 1.62 (1.28, 2.05) | <0.001 |
| 25(OH)D (nmol/L) | 66.59 ± 28.57 | 0.99 (0.98, 0.99) | <0.001 |
| 25(OH)D (nmol/L) Z score | -0.00 ± 1.00 | 0.69 (0.60, 0.79) | <0.001 |
| Hypercholesterolemia |  |  |  |
| no | 1823 (62.26%) | Reference |  |
| yes | 1091 (37.26%) | 0.81 (0.63, 1.04) | 0.095 |
| Diabetes mellitus |  |  |  |
| no | 2591 (88.55%) | Reference |  |
| yes | 335 (11.45%) | 1.22 (0.86, 1.72) | 0.267 |
| Glycohemoglobin (%) | 5.74 ± 1.03 | 1.20 (1.10, 1.32) | <0.001 |
| Fasting Glucose (mmol/L) | 5.95 ± 1.81 | 1.09 (1.01, 1.17) | 0.031 |
| Direct HDL-Cholesterol (mmol/L) | 1.38 ± 0.42 | 0.69 (0.51, 0.94) | 0.018 |
| Health insurance |  |  |  |
| no | 601 (20.53%) | Reference |  |
| yes | 2326 (79.47%) | 0.46 (0.36, 0.60) | <0.001 |
| Smoking status |  |  |  |
| Never smoking | 1718 (58.71%) | Reference |  |
| Former smoking | 431 (14.73%) | 4.09 (3.07, 5.46) | <0.001 |
| Current smoking | 777 (26.56%) | 1.79 (1.35, 2.39) | <0.001 |
| Total Cholesterol( mmol/L) | 5.03 ± 1.09 | 0.94 (0.84, 1.05) | 0.294 |
| Triglyceride (mmol/L) | 1.44 ± 1.69 | 0.94 (0.80, 1.10) | 0.429 |
| LDL-cholesterol (mmol/L) | 2.99 ± 0.90 | 1.06 (0.88, 1.28) | 0.547 |

BMI, body mass index; AA, associate degree; GED, General Educational Development; HDL-Cholesterol, high-density lipoprotein cholesterol; LDL-Cholesterol, low-density lipoprotein cholesterol;

**Supplement Table 3**. subgroup analysis for the interaction of 25(OH)D levels in different group

| Variables | N | OR, 95%CI | P for interaction |
| --- | --- | --- | --- |
| Gender |  |  | 0.327 |
| Male | 1371 | 0.99 (0.98, 0.99) |  |
| Female | 1464 | 0.99 (0.98, 1.00) |  |
| Age(year) group |  |  | 0.867 |
| 30 - 40 | 854 | 0.98 (0.97, 1.00) |  |
| 41 - 55 | 1017 | 0.98 (0.97, 0.99) |  |
| 56 - 80 | 964 | 0.98 (0.97, 0.99) |  |
| Race/Hispanic origin |  |  | 0.003 |
| Mexican American | 415 | 1.01 (1.00, 1.02) |  |
| Other Hispanic | 242 | 1.00 (0.99, 1.02) |  |
| Non-Hispanic White | 1242 | 0.98 (0.97, 0.99) |  |
| Non-Hispanic Black | 499 | 0.99 (0.98, 1.00) |  |
| Other Race | 437 | 1.00 (0.98, 1.01) |  |
| Education level |  |  | 0.260 |
| Less than 11th grade | 516 | 1.00 (0.99, 1.00) |  |
| High school graduate/GED | 576 | 0.99 (0.98, 1.00) |  |
| Some college or AA degree | 847 | 0.98 (0.97, 0.99) |  |
| College graduate or above | 895 | 0.99 (0.97, 1.00) |  |
| Marital status |  |  | 0.749 |
| Single/divorced/widowed | 594 | 0.99 (0.98, 0.99) |  |
| Never married | 322 | 0.99 (0.97, 1.00) |  |
| Married/living as married | 1918 | 0.99 (0.98, 1.00) |  |
| Ratio of family income to poverty |  |  | 0.153 |
| <1.3 | 727 | 0.99 (0.99, 1.00) |  |
| 1.3-3.5 | 874 | 0.99 (0.98, 1.00) |  |
| >3.5 | 1014 | 0.98 (0.97, 0.99) |  |
| Alcohol consumption |  |  | <0.01 |
| no | 677 | 1.00 (0.99, 1.01) |  |
| yes | 1998 | 0.98 (0.98, 0.99) |  |
| Apolipoprotein (B) (mg/dL) group |  |  | 0.992 |
| < 81 | 450 | 0.98 (0.97, 1.00) |  |
| 82 - 101 | 447 | 0.98 (0.97, 1.00) |  |
| >102 | 454 | 0.98 (0.97, 0.99) |  |
| BMI(Kg/m2) group |  |  | 0.603 |
| < 25.7 | 918 | 0.99 (0.98, 1.00) |  |
| 25.8 - 30.8 | 956 | 0.98 (0.98, 0.99) |  |
| >30.9 | 948 | 0.99 (0.98, 1.00) |  |
| Hypertension |  |  | 0.387 |
| no | 1833 | 0.98 (0.98, 0.99) |  |
| yes | 1001 | 0.99 (0.98, 0.99) |  |
| Diabetes mellitus |  |  | 0.030 |
| no | 2511 | 0.98 (0.98, 0.99) |  |
| yes | 322 | 1.00 (0.99, 1.01) |  |
| Glycohemoglobin (%) group |  |  | 0.469 |
| <5.2 | 706 | 0.98 (0.97, 0.99) |  |
| 5.3 - 5.6 | 1062 | 0.99 (0.98, 1.00) |  |
| >5.7 | 1065 | 0.99 (0.98, 1.00) |  |
| Fasting Glucose (mmol/L) group |  |  | 0.783 |
| <5.22 | 406 | 0.98 (0.97, 1.00) |  |
| 5.27 - 5.77 | 474 | 0.98 (0.97, 0.99) |  |
| >5.83 | 472 | 0.99 (0.97, 1.00) |  |
| Health insurance |  |  | 0.031 |
| no | 580 | 1.00 (0.99, 1.01) |  |
| yes | 2254 | 0.99 (0.98, 0.99) |  |
| Smoking status |  |  | 0.915 |
| Never smoking | 1668 | 0.99 (0.98, 1.00) |  |
| Former smoking | 416 | 0.99 (0.98, 1.00) |  |
| Current smoking | 749 | 0.99 (0.98, 1.00) |  |
| Total Cholesterol (mmol/L) group |  |  | 0.691 |
| < 4.53 | 938 | 0.99 (0.98, 1.00) |  |
| 4.55 - 5.38 | 947 | 0.99 (0.98, 1.00) |  |
| >5.4 | 943 | 0.98 (0.98, 0.99) |  |
| Triglyceride (mmol/L) group |  |  | 0.468 |
| < 0.85 | 445 | 0.98 (0.96, 0.99) |  |
| 0.86 - 1.39 | 455 | 0.99 (0.98, 1.00) |  |
| >1.4 | 451 | 0.98 (0.97, 0.99) |  |
| LDL-cholesterol (mmol/L) group |  |  | 0.985 |
| <2.53 | 432 | 0.98 (0.97, 1.00) |  |
| 2.56 - 3.26 | 435 | 0.98 (0.97, 1.00) |  |
| > 3.28 | 456 | 0.98 (0.97, 0.99) |  |

BMI, body mass index; AA, associate degree; GED, General Educational Development; HDL-Cholesterol, high-density lipoprotein cholesterol; LDL-Cholesterol, low-density lipoprotein cholesterol;

**Supplementary materials**

1. The definition of smoking status was classified as “current smoking,” “former smoking,” and “never smoking,” which were defined based on answers from two questions: “Have you smoked at least 100 cigarettes in your life?” and “Do you now smoke cigarettes?”.

2. Alcohol consumption was defined as having at least 12 drinks of any type of alcoholic beverage in the past year.
